# Human fallopian tube: a new source of multipotent adult mesenchymal stem cells discarded in surgical procedures

**DOI:** 10.1186/1479-5876-7-46

**Published:** 2009-06-18

**Authors:** Tatiana Jazedje, Paulo M Perin, Carlos E Czeresnia, Mariangela Maluf, Silvio Halpern, Mariane Secco, Daniela F Bueno, Natassia M Vieira, Eder Zucconi, Mayana Zatz

**Affiliations:** 1 Human Genome Research Center, Biosciences Institute, University of São Paulo, Brazil Rua do Matão, n° 106, Cidade Universitária São Paulo SP, CEP: 05508-090, Brazil; 2 CEERH Specialized Center for Human Reproduction, São Paulo, Brazil Rua Mato Grosso, n° 306 19° andar, Higienópolis São Paulo SP, CEP: 01239-040, Brazil; 3 Celula Mater, São Paulo, Brazil Al. Gabriel Monteiro da Silva, n° 802 São Paulo SP, CEP: 01442-000, Brazil

## Abstract

**Background:**

The possibility of using stem cells for regenerative medicine has opened a new field of investigation. The search for sources to obtain multipotent stem cells from discarded tissues or through non-invasive procedures is of great interest. It has been shown that mesenchymal stem cells (MSCs) obtained from umbilical cords, dental pulp and adipose tissue, which are all biological discards, are able to differentiate into muscle, fat, bone and cartilage cell lineages. The aim of this study was to isolate, expand, characterize and assess the differentiation potential of MSCs from human fallopian tubes (hFTs).

**Methods:**

Lineages of hFTs were expanded, had their karyotype analyzed, were characterized by flow cytometry and underwent *in vitro *adipogenic, chondrogenic, osteogenic, and myogenic differentiation.

**Results:**

Here we show for the first time that hFTs, which are discarded after some gynecological procedures, are a rich additional source of MSCs, which we designated as *human tube MSCs *(htMSCs).

**Conclusion:**

Human tube MSCs can be easily isolated, expanded *in vitro*, present a mesenchymal profile and are able to differentiate into muscle, fat, cartilage and bone *in vitro*.

## Background

Adult mesenchymal stem cells (MSCs) are typically defined as undifferentiated multipotent cells endowed with the capacity for self-renewal and the potential to differentiate into several distinct cell lineages [1]. These progenitor cells which constitute a reservoir found within the connective tissue of most organs are involved in the maintenance and repair of tissues throughout the postnatal life of an individual. Although functionally heterogeneous, MSC populations isolated from different tissues such as bone marrow, skeletal muscle, lung, adipose tissue, dental pulp, placenta, and the umbilical cord present a similar profile of cell surface receptor expression [2-10]. However, it is also well known that adult stem cells are defined by their functional properties rather than by marker expression [11].

We and others have recently shown that the umbilical cord, dental pulp, orbicular oris muscle and adipose tissue are a very rich source of MSCs able to differentiate into muscle, cartilage, bone and adipose cell lineages [7,10,12-15]. The extraordinary regenerative capacity of the human endometrium following menstruation, in the postpartum period, after surgical procedures (uterine curettage, endometrial ablation) and in postmenopausal women undergoing hormonal replacement therapy suggests that MSC niches present in this tissue could be responsible for this process [16]. Indeed, endometrial and menstrual blood-derived stem cells were recently isolated and showed the ability to differentiate into cell types of the three germ layers [17-23].

The human fallopian tubes (hFTs) share the same embryologic origin as the uterus. They have the capacity to undergo dynamic endocrine-induced changes during the menstrual cycle, including cell growth and regeneration, in order to provide the unique environment required for the maintenance of male and female gamete viability, fertilization, and early embryo development as well as transport to the uterus [24]. Therefore, based on the experience of our research group in the identification, and characterization of potential sources of adult stem cells [7,10,12-15], the aim of this study was to isolate, expand, characterize and assess the differentiation potential of MSCs from hFTs.

## Methods

### Human Fallopian Tube Collection and Processing

Human fallopian tubes (*n *= 6) were obtained from hysterectomy or tubal ligation/resection samples collected during the proliferative phase from fertile women in their reproductive years (range 35–53 years) who had not undergone exogenous hormonal treatment for at least three months prior to surgery. Informed consent was obtained from each patient and approval granted from by the ethics committee of the Biosciences Institute of the University of São Paulo. All laboratory experiments were carried out at the Human Genome Research Center, São Paulo, Brazil.

Each sample was collected in HEPES-buffered Dulbecco Modified Eagle Medium/Hams F-12 (DMEM/F-12; Invitrogen, Carlsbad, CA) or DMEM high glucose (DMEM/High; Invitrogen, Carlsbad, CA) supplemented with 10% fetal bovine serum (FBS; HyClone, Logan, UT), kept in 4°C and processed within 24 hours period. All hFTs samples were washed twice in phosphate saline buffer (PBS, Gibco, Invitrogen, Carlsbad, CA), finely minced with a scalpel, put inside a 15 or 50 mL falcon, and incubated in 5 ml of pure TripLE Express, (Invitrogen, Carlsbad, CA n) for 30 minutes, at 37°C, in a water bath. Subsequently, supernatant was removed with a sterile Pasteur pipette, washed once with 7 mL of DMEM/F-12 supplemented with 10% FBS in a 15 mL falcon, and pelleted by centrifugation at 400 g for five minutes at room temperature. Cells were then plated in DMEM/F-12 (5 mL) supplemented with 10% FBS, 100 IU/mL penicillin (Invitrogen) and 100 IU/mL streptomycin (Invitrogen, Carlsbad, CA) in plastic flasks (25 cm^2^), and maintained in a humidified atmosphere of 5% CO_2 _in air at 37°C. The culture medium used for expansion was initially changed every 72 hours and routinely replaced twice a week thereafter.

### Population Doubling (PD) and Karyotypic Analysis

PD experiments were carried out to verify the growth rate of cell lineages for at least five consecutive days, both during the process of establishment and long-term passages. To calculate the growth rate the methodology previously described by Deasy *et al. *was used [25].

Karyotypic analysis of cells from the same lineages undergoing PD experiments was performed to verify maintenance of chromosomal normality. Cells were cultured for one hour in colchicine (0.1 μg/mL), detached using TripLE Express (Invitrogen, Carlsbad, CA), washed in PBS (Gibco – Invitrogen, Carlsbad, CA), and resuspended in 0.5 mL of medium and mixed with .075 M KCl to a volume of 10 mL. After incubation for 20 minutes at 37°C in a water bath, the cells were centrifuged at 400 g for five minutes and the pellet fixed three times in 1 mL of cold Carnoy's fixative. Three drops of cell suspension were fixed per slide. For chromosome counting the slides were stained in Giemsa for 15 minutes and photographed in a phase-contrast microscope (Ikaros System, Axiophot 2, Carl Zeiss, Jena, Germany)

### Flow Cytometry Analysis

Flow cytometry analysis was performed using a Guava EasyCyte microcapillary flow cytometer (Guava Technologies, Hayward, CA) utilizing laser excitation and emission wavelengths of 488 and 532 nm, respectively. Cells were pelleted, resuspended in PBS (Gibco – Invitrogen, Carlsbad, CA) at a concentration of 1.0 × 10^5 ^cells/mL and stained with saturating concentration of antibodies. After 45 minute incubation in the dark at room temperature, cells were washed three times with PBS (Gibco, Invitrogen, Carlsbad, CA) and resuspended in 0.25 mL of cold PBS.

In order to analyze cell surface expression of typical protein markers, adherent cells were treated with the following anti-human primary antibodies: CD13-phycoerythrin [PE] (Becton Dickinson, Franklin Lakes, NJ), CD14 (VMRD Inc., Pullman, WA), CD29-PE-Cy5, CD31-PE, CD34-PerCP, CD38-fluorescein isothiocyanate [FITC], CD44-FITC, CD45-FITC, CD73-PE, CD90-R-PE, CD117-PE (Becton Dickinson, Franklin Lakes, NJ), CD133-PE (Miltenyi Biotec, Gladbach, Germany), human leukocyte antigens (HLA)-ABC-FITC and HLA-DR-R-PE (Becton Dickinson, Franklin Lakes, NJ), SSEA4 (Chemicon, Temecula, CA), STRO1 (R&D Systems, Minneapolis, MN), and SH2, SH3 and SH4 (kindly provided by Dr. Kerkis, Butantan Institute, São Paulo, Brazil). Unconjugated markers were reacted with anti-mouse PE secondary antibody (Guava Technologies, Hayward, CA). Unstained cells were gated on forward scatter to eliminate particulate debris and clumped cells. A minimum of 5.000 events were counted for each sample.

### Mesenchymal Stem Cell Differentiation

To evaluate the properties of mesenchymal stem cell differentiation, adherent cells (3^rd ^and 11^th ^passages) underwent *in vitro *adipogenic, chondrogenic, osteogenic, and myogenic differentiation according to the following protocols:

#### Adipogenic Differentiation

The adipogenic differentiation capacity of culture-expanded hFTs cells was determined as previously reported [26]. Cultured-expanded cells from hFTs were cultured in proliferation medium supplemented with 1 μM dexamethasone, 500 μM 3-isobutyl-1-methylxanthine, 60 μM indomethacin, and 5 μg/mL insulin (Sigma-Aldrich, St. Louis, MO). Confirmation of adipogenic differentiation was obtained on day 21 by intracellular accumulation of lipid-rich vacuoles stainable with oil red O (Sigma-Aldrich, St. Louis, MO). For the oil red O stain cells were fixed with 4% paraformaldehyde (PFA) for 30 minutes, washed, and stained with a working solution of 0.16% oil red O for 20 minutes.

#### Chondrogenic Differentiation

Approximately 2.5 × 10^5 ^hFTs were centrifuged in a 15 mL polystyrene tube at 500 g for five minutes, and the pellet resuspended in 10 mL of basal medium. The basal medium consisted of DMEM/High (Invitrogen, Carlsbad, CA) supplemented with 1% ITS-Premix (Becton Dickinson, Franklin Lakes, NJ), 1% 10 mM dexamethasone (Sigma-Aldrich, St. Louis, MO), 1% 100 mM sodium pyruvate (Gibco – Invitrogen, Carlsbad, CA), and 1% 5 mM ascorbic acid-2 phosphate (Sigma-Aldrich, St. Louis, MO). Without disturbing the pellet, cells were resuspended in 0.5 mL of chondrogenic differentiation medium, consisting of the basal medium supplemented with 10 ng/mL transforming growth factor (TGF) β1 (R&D Systems, Minneapolis, MN) and 10% FBS, maintained in a humidified atmosphere of 5% CO_2 _in air at 37°C.

On day one, tubes were gently turned over to acquire a single floating cell sphere. Medium was changed every three or four days. On day 21, samples were fixed in 10% formalin for 24 hours at 4°C and paraffin-embedded. Cryosections (5 μm thick) were cut from the harvested micromasses and stained with toluidine blue to demonstrate extracellular matrix mucopolysaccharides [14].

#### Osteogenic Differentiation

Osteogenic differentiation was obtained by culturing hFTs cells in DMEM low glucose (DMEM/LG; Invitrogen, Carlsbad, CA) supplemented with 0.1 mM dexamethasone and 50 mM ascorbic acid-2 phosphate (both Sigma-Aldrich, St. Louis, MO) and maintained in a humidified atmosphere of 5% CO_2 _in air at 37°C. On day nine, 10 mM β-glycerolphosphate was added to induce mineralization. Osteogenic differentiation was shown by formation of calcium-hydroxyapatite-positive areas (von Kossa staining) on day 21. After two washes with PBS (Gibco – Invitrogen, Carlsbad, CA) and one with distilled water, the cells were incubated in 1% silver nitrate (Sigma-Aldrich, St. Louis, MO) under ultraviolet light for 45 minutes. The cells were then incubated in 3% sodium thiosulfate (Sigma-Aldrich, St. Louis, MO) for 5 minutes. Counterstaining was finally performed with Van Gieson [14]. The calcium accumulation was indicated by dark color.

#### Myogenic Differentiation

For myogenic differentiation hFTs cells were cultured in myogenic differentiation medium consisting of 50% induction medium and 50% fresh DMEM/F-12 (Invitrogen, Carlsbad, CA) supplemented with 10% FBS (HyClone, Logan, UT) in a humidified atmosphere of 5% CO_2 _in air at 37°C.

Proliferation medium, which consists of DMEM/F-12 supplemented with 10% FBS, 100 IU/mL penicillin (Invitrogen, Carlsbad, CA), and 100 IU/mL streptomycin (Invitrogen, Carlsbad, CA), is in fact the same medium used previously to cultivate primary human myoblasts for 48 hours. Prior to its use, induction medium was filtered through a 0.22 μm pore membrane filter (Millipore, Billerica, MA) and pH was adjusted with sodium bicarbonate (Sigma-Aldrich, St. Louis, MO). The hFTs MSCs were cultured for 40 days and the medium changed twice a week. After this interval, cells were analyzed using Immunofluorescence (IF) and Western blot (WB) testing.

### Immunofluorescence and Western Blot Analysis

#### Immunofluorescence (IF)

Immunofluorescence localization of dystrophin was performed on muscle-differentiated hFTs cells to confirm myogenic differentiation. Cells were washed twice with cold PBS (Gibco – Invitrogen, Carlsbad, CA), fixed with 4% PFA/PBS for 20 minutes at 4°C, and permeabilized with .05% Triton X-100 (TX-100; Sigma-Aldrich, St. Louis, MO) in PBS (Gibco – Invitrogen, Carlsbad, CA) for five minutes. After blocking non-specific binding 10% FBS/PBS (Invitrogen, Carlsbad, CA) for one hour at room temperature, incubations with the primary antibody (anti-dystrophin; Ab15277; Abcam, Cambridge, UK) overnight at 4°C and the secondary antibody (FITC IgG; Chemicon, Temecula, CA) for one hour at room temperature were performed. Nuclei were counterstained with 4',6-diamidino-2-phenylindole (DAPI; Sigma-Aldrich, St. Louis, MO) for visualization. As positive controls, we used normal human differentiated myotubes cultures. As negative controls, we used non-diferentiated htMSCs. The immunofluorescence slides were examined using an Axiovert 200 microscope (Axio Imager Z1, Carl Zeiss, Oberkochen, Germany).

#### Western Blot

Proteins of muscle-differentiated hFTs cells were extracted by treatment with a buffer containing 10 mM Tris-HCL [pH 8.0], 150 mM Nacl, 5 mM EDTA, 1% TX-100, and 60 mM octyl glucoside (Sigma-Aldrich, St. Louis, MO). Samples were centrifuged at 13.000 g for 10 minutes to remove insoluble debris. Proteins were separated by sodium dodecyl sulfate-polyacrylamide gel electrophoresis (SDS-PAGE 6%) and transferred onto nitrocellulose membranes (Amersham Biosciences, Piscataway, NJ). All membranes were stained with 0.2% Ponceau S (Sigma-Aldrich) to evaluate the amount of loaded proteins. Membranes were blocked for one hour at room temperature with 5% milk powder in Tris-buffered saline with Tween 20 detergent (TBST, 20 mM Tris-HCL, 500 mM NaCl, .05% Tween 20) and treated overnight with anti-dystrophin (VP-D508; Vector Laboratories, Burlingame, CA) and anti-skeletal myosin (M7523; Sigma-Aldrich, St. Louis, MO) primary antibodies. The following day, membranes were incubated for one hour at room temperature with peroxidase-conjugated anti-mouse and anti-rabbit IgG secondary antibodies (GE Healthcare, Piscataway, NJ) as recommended by the manufacturer. Immunoreactive bands were detected using the Enhanced Chemoluminescence Detection System (GE Healthcare, Piscataway, NJ).

## Results

### Lineages Expansion, Population Doubling (PD) and Karyotype analysis

**Figure 1:**
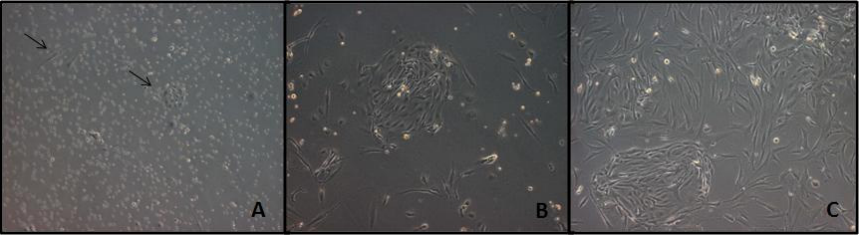
**Morphology of adherent cells when isolated from hFTs (primary cultures)**. A): Cells cultured for three days after initial plating. Cells with an MSC-like phenotype and a small cluster of cells with endothelial appearance (arrows) (100×). B): Cells cultured for six days after initial plating (100×). C) Cells cultured for six days after initial plating (400×). (Microscope Zeiss Axiovert 200).

**Figure 2:**
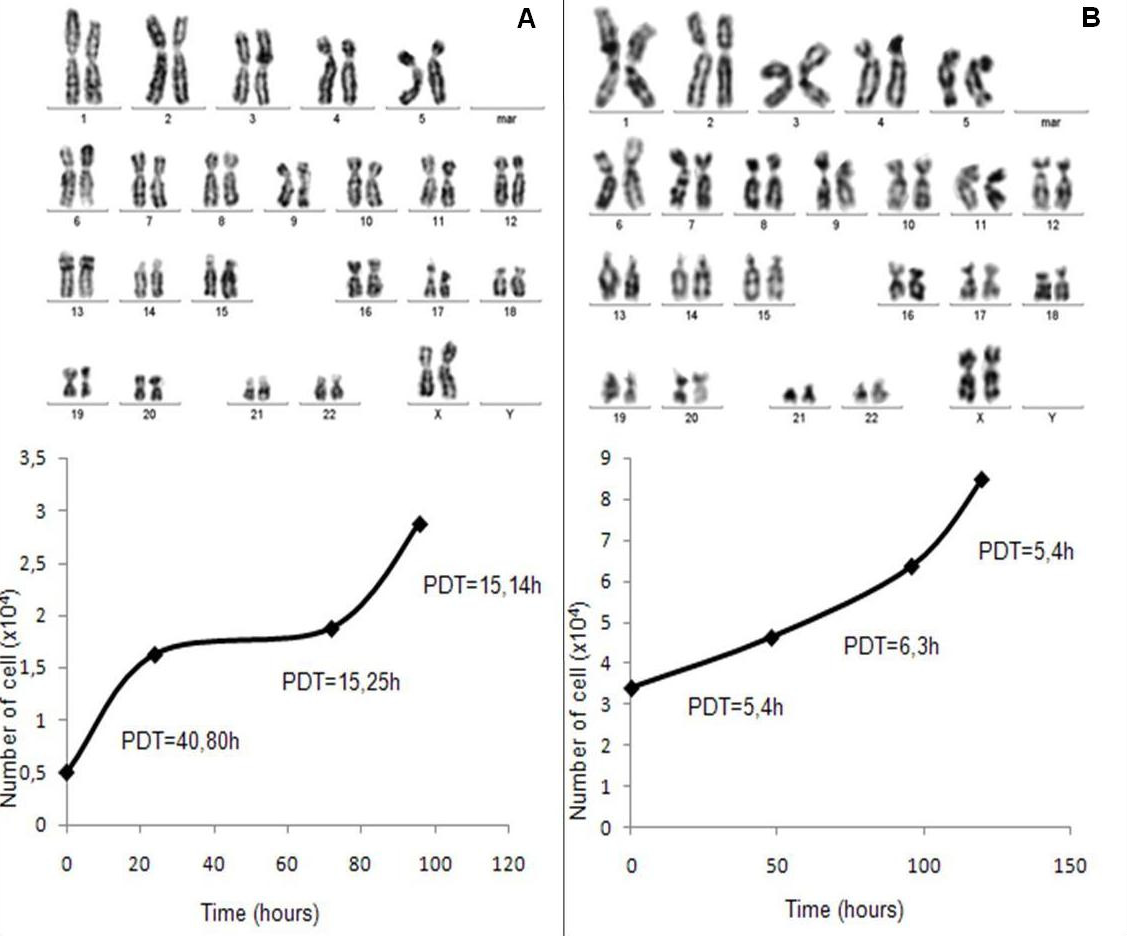
**Population doubling and karyotypic analysis**. Panel A) Results of hFTs lineage in passage two. Panel B) Results of hFTs lineage in passage 11. We observed high rates of cell division, with gradual decreasing of the population doubling time (PDT) in lineages cultured for a long time. Despite that, no evidence of chromosomal abnormality was observed (Ikaros System, Axiophot 2, Carl Zeiss).

After plating hFTs cells, different cell types were observed but most were spindle-shaped, resembling fibroblasts. Some clusters of cells with endothelial appearance, which spread weakly, could also be observed (figure 1). After the first enzymatic dissociation, usually between 5–7 days of culture, adherent cells were constituted of homogeneous cell layers with a MSC-like phenotype. All lineages were expanded, frozen and thawed several times. PD experiments showed high rates of cell division and karyotypic analysis showed no evidence of chromosomal abnormalities (figure 2).

### Flow Cytometry Analysis

All adherent cells derived from hFTs did not express hematopoietic lineage markers (CD34, CD38, CD45, CD117 and CD133), endothelial marker CD31 and monocyte marker (CD14). In addition, the majority of cells expressed high levels of adhesion markers (CD29, CD44 and CD90) and MSCs markers (CD13, CD73, SH2, SH3 and SH4). The isolated cells from hFTs were also positive for HLA-class I (HLA-ABC) but negative for HLA-class II (HLA-DR), and negative as well for the embryonic stem cell factor SSEA4 and the presumed MSC marker Stro1.

**Figure 3:**
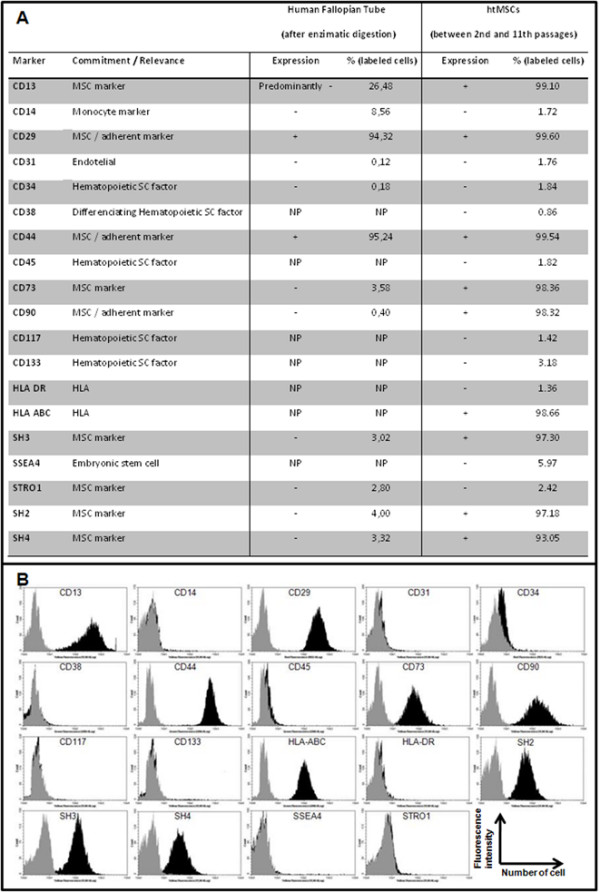
**Cytometry analysis of htMSCs**. Panel A) Analyzed markers, its commitment, its expression (positive or negative) in fresh digested hFTs and in htMSCs, and the mean percentage of positive labeled cells and analyzed by flow cytometry (GuavaTechnologies, Hayward, CA, ). NP means "not performed". Panel B) Related graphs, where it is possible to compare, for each of the 19 analyzed markers, the control sample (not labeled htMSCs) in gray and the experimental population of htMSCs (labeled with specific antibodies) in black.

For a comparative investigation, we provided a cytometry analysis of freshly digested and not cultured hFT, where we used 9 mesenchymal stem cells markers (CD13, CD29, CD44, CD73, CD90, Stro-1, SH2, SH3 and SH4), as well as tissue specific markers (CD14, CD31 and CD34). The cytometry analysis summarized in figure 3 shows the mesenchymal profile for hFTs cells. Additionally, MSC properties of isolated cells were further confirmed with cell differentiation studies. Surprisingly, CD29 and CD44 were positively expressed in htMSC and in freshly digested and not cultured hFTs.

### Multilineage Differentiation

**Figure 4:**
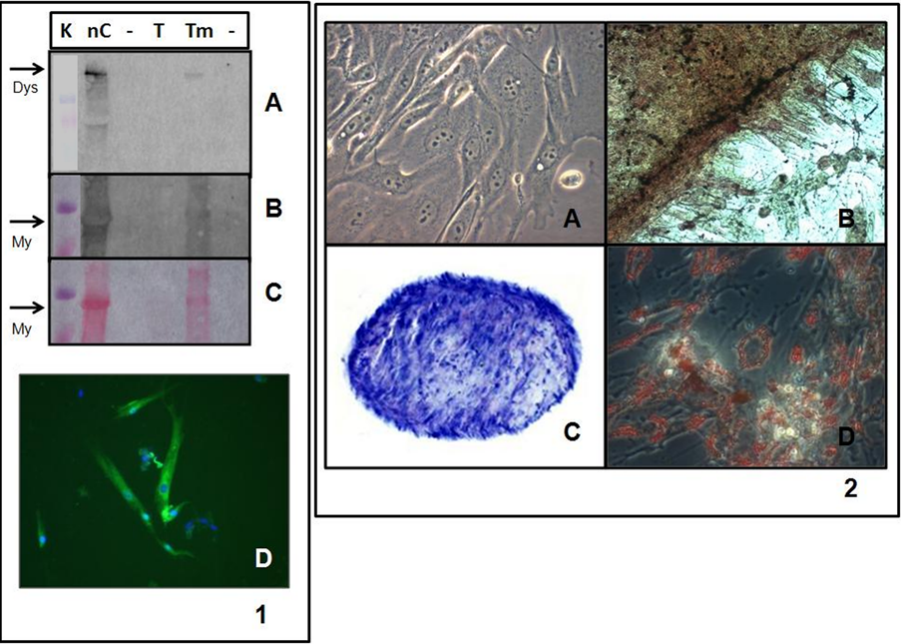
**Multilineage differentiation *in vitro***. Panel 1) Myogenic differentiation. *K *represents Kaleidoscope (BioRad, molecular marker), *nC *represents normal control of human skeletal muscle, *T *represents htMSCs control, *Tm *represents hFTs cells induced for myogenic differentiation. 1A) Dystrophin expression in muscle control and in the induced hFTs cells. 1B) Skeletal myosin expression in muscle control and in the induced hFTs cells. 1C) Myosin band observed in the muscle control and in the induced hFTs cells by Ponceau S membrane dyeing. 1D) IF assay, indicating dystrophin expression (in green fluorescence) in myotubes differentiated from hFTs cells, where nucleuses were colored with DAPI (blue fluorescence) (400×). Panel 2) Osteogenic, chondrogenic and adipogenic differentiation of hFTs cells. 2A) Control hFTs cells (630×). 2B) Osteogenic differentiation (200×). 2C) Chondrogenic differentiation (100×). 2D) Adipogenic differentiation (630×) (Microscope Zeiss Axiovert 200).

The plasticity of adherent cells obtained from hFTs was assessed three weeks after mesodermal induction for osteogenic, adipogenic, and chondrogenic differentiation. The multilineage differentiation was performed for 5 independent lineages of htMSCs, and no evident difference in their differentiation potential was observed between them. In addition, the potential for hFTs cells to differentiate into skeletal muscle cells was investigated after 40 days of culture in induction medium. The myogenic differentiation was demonstrated by the expression of myogenic markers (myosin and dystrophin). The hFTs cells differentiated in myogenic, adipogenic, chondrogenic, and osteogenic tissues *in vitro *(figure 4). Together, these results confirmed the mesenchymal nature of the isolated cells and their multipotency.

## Discussion

The possibility of using stem cells for regenerative medicine has opened a new field of investigation to find the best sources for obtaining multipotent stem cells, in particular through non-invasive procedures.

Initially defined as bone marrow precursors, new evidence suggests that MSCs are present in virtually all organs playing a possibly important role in tissue maintenance and regeneration [27-31]. More recently, they were also found in the human uterus endometrium and in menstrual blood and have been shown capable of promoting regeneration *in vivo *[16,18,19,21,22,32,33]. A recent study demonstrated isolating stem cells from the endometrium and promoting *in vitro *chondrogenesis [20].

It has been shown that MSCs obtained from the umbilical cord, dental pulp, adipose tissue and menstrual blood, all biological discards, are able to differentiate into muscle, fat, bone and cartilage cell lineages [7,10,12-15]. Here we show for the first time that the hFTs, which are discarded in hysterectomy procedures, are an additional source rich in MSCs, which we designated as *human tube MSCs *(htMSCs). Early passage htMSCs had longer PD times (approximately 15 hours). However, with additional passages, PD times shortened and stabilized. Although htMSCs proliferate extensively in culture, comparative analysis of the cells' karyotypes from early (second) and late (eleventh) passages showed no abnormalities, suggesting chromosomal stability throughout the passages.

Although Nasef *et al. *suggest that a purified Stro-1-enriched population augment the suppressive effect in allogeneic transplantation, Murphy *et al. *showed that allogeneic endometrial regenerative cells (ERC or menstrual blood mesenchymal stem cells), that are Stro-1 negative, were efficient for the treatment of critical limb ischemia in rats [34,32]. In accordance to recent studies in human endometrium, htMSCs are also Stro1 negative [35]. In other hand, CD44, which is considered a marker of MSCs and has been shown to be critical for the recruitment of MSCs into wound sites for tissue regeneration, was highly expressed in htMSCs and also in the fresh digested fallopian tube tissue [36,37]. CD29, an integrin involved in cell adhesion was also greatly expressed in all htMSCs studied lineages, including freshly digested samples. Curiously, according to evidences from recent studies, this molecule may be involved in the fertilization process, allowing the binding and fusion of sperm and egg [38].

However, speculation that htMSCs may play a role in reproduction remains to be elucidated. Anyway, the high levels of expression of adhesion markers (CD29, CD44 and CD90) and other MSC markers (CD13, CD73, SH2, SH3 and SH4) together with the multilineage differentiation results confirmed the mesenchymal nature of human fallopian tube stem cells. These important features imply that htMSCs represent a cell population that can be rapidly expanded for potential clinical applications.

The morphological and functional integrity of the tubal epithelium are of paramount importance for the development of a unique microenvironment required for optimal fertilization and early embryo development. They are therefore essential for successful implantation as evidenced by a recent meta-analysis showing that the use of human oviductal cells for co-culture improves embryo morphology, implantation rates and pregnancy success [39].

Anatomically the hFTs are divided into four distinct segments (intramural, isthmic, ampulla, and infundibulum/fimbria) each one comprised of different populations of epithelial cells and distinct secretory activity [40]. Bacteria and viruses constantly found in the lumen of the vagina may sporadically enter the upper reproductive tract disrupting the hFTs epithelial integrity, and represent a significant risk factor to female reproductive health. The need of a strict homeostasis of hFT environment in order to avoid the disruption of the reproductive function suggests that MSC niches present in this tissue could be responsible for this process [41,42].

Recently, Wolff *et al. *were able to demonstrate the presence of endometrial multipotent cells by inducing chondrogenic differentiation *in vitro *of a subpopulation of endometrial stromal cells [20]. However, using non-endometrial gynecologic tissue such as myometrium, fallopian tube, and uterosacral ligaments as controls, they could not demonstrate chondrogenesis. This suggests that there may be less progenitor stem cells in these tissues due to their lower burden of lifelong regeneration compared with the endometrium; or that the differentiation assay employed in their study was not appropriate for these tissues. Based on our success in obtaining myogenic, adipogenic, osteogenic, and chondrogenic differentiation from htMSCs we may presume that the inability to demonstrate chondrogenesis from fallopian tube tissue reported by Wolff *et al. *could be related to methodological issues rather than to progenitor stem cell concentration.

## Conclusion

Human tissue fragments that are usually discarded in surgical procedures may represent important sources of stem cells and their use does not pose ethical problems. This is the first study to demonstrate the isolation, *in vitro *expansion, and differentiation into muscle, fat, cartilage, and bone of a new rich source of mesenchymal progenitor cells from normal adult hFTs. Tissue fragments of hFTs, which are usually discarded after surgical procedures, may represent a new potential source of pluripotent cells for regenerative medicine. The identification of niches of tissue-specific stem cells capable of replacing damaged differentiated cells in the hFTs may contribute to provide the unique environment required for the maintenance of male and female gamete viability, fertilization, and early embryo development and transport to the uterus, altogether necessary for a successful reproductive outcome.

## Competing interests

The authors declare that they have no competing interests.

## Authors' contributions

TJ and MZ conceived the study. PMP, CEC, MM and SH provide human tubes from surgical procedures. TJ, MZ, PMP, CEC, MM and SH wrote the manuscript. TJ designed and performed tissue cultures, Western Blotting and Immunofluorescence. MS, EZ and NMV helped with flow cytometric evaluation and with the manuscript review. DFB helped with osteogenic and chondrogenic differentiation. All authors read and approved the final manuscript.
